# Learning about phraseology from corpora: A linguistically motivated approach for Multiword Expression identification

**DOI:** 10.1371/journal.pone.0237767

**Published:** 2020-08-27

**Authors:** Uxoa Inurrieta, Itziar Aduriz, Arantza Díaz de Ilarraza, Gorka Labaka, Kepa Sarasola

**Affiliations:** 1 HiTZ Basque center for language technologies, Ixa NLP group, University of the Basque Country, Leioa, Spain; 2 Department of Catalan Philology and General Linguistics, University of Barcelona, Barcelona, Spain; STL UMR8163 CNRS, FRANCE

## Abstract

Multiword Expressions (MWEs) are idiosyncratic combinations of words which pose important challenges to Natural Language Processing. Some kinds of MWEs, such as verbal ones, are particularly hard to identify in corpora, due to their high degree of morphosyntactic flexibility. This paper describes a linguistically motivated method to gather detailed information about verb+noun MWEs (VNMWEs) from corpora. Although the main focus of this study is Spanish, the method is easily adaptable to other languages. Monolingual and parallel corpora are used as input, and data about the morphosyntactic variability of VNMWEs is extracted. This information is then tested in an identification task, obtaining an F score of 0.52, which is considerably higher than related work.

## Introduction

Multiword Expressions (MWEs) are combinations of words which exhibit some kind of lexical, morphosyntactic, semantic, pragmatic or statistical idiosyncrasy [1]. Due to their idiosyncratic nature, they pose multiple challenges to Natural Language Processing (NLP), and sophisticated strategies are needed in order to process them correctly.

Several types of word combinations are comprised in the category of MWEs [2, 3], such as idioms (example 1), which have a non-compositional meaning, and collocations, where the lexical choice is restricted (example 2). The latter also include light verb constructions (example 3), where the verb tends to be semantically bleached. In the examples in this paper, lexicalised component words of MWEs [4] are bold, and other words or morphemes that need to be marked are underlined. When glosses are given, the Leipzig glossing rules and abbreviations are used.

(1) *She always ends up* ***spilling the beans*** (lit. revealing the secret)
(2) *All students* ***passed*** *the* ***exam***.
(3) *She is* ***giving*** *a* ***lecture*** *this afternoon*.

Two of the most challenging features of MWEs are variability and discontiguity [5], that is, the fact that the component words of many MWEs can occur in several word forms, can be separated by other elements in a sentence, and can even have an inverted word order. These variations are especially prominent in MWEs where the syntactic head is a verb, since combinations of these kinds tend to be rather flexible morphosyntactically (example 4). However, many such combinations are not completely flexible and have some restrictions (example 5).

(4)
  a *They* ***made*** *a* ***conclusion***.
  b *They* ***made*** *some simple but still interesting* ***conclusions***.
  c *The* ***conclusions*** *they* ***make*** *are always interesting*.
(5)
  a *Their advice should be* ***taken into account***.
  b *You should* ***take*** *their advice* ***into account***.
  c *The accounts into which their advice should be taken.

Therefore, for the identification of verbal MWEs, basic methods which try to match fixed word sequences against dictionary entries are too limited. Let *make conclusions* and *take into account* be two entries in a dictionary. If this basic method was employed to identify occurrences of these entries in the sentences in examples (4) and (5a)–(5b), all occurrences would be ignored, because: the component words are separated by external elements in (4a)–(4c) and (5b); word forms in examples (4b), (4c) and (5a) are different than the ones in the entry; and word order is inverted in example (4c).

On the other hand, opposite strategies where only the lemmas of the component words are searched for (within a given word distance) are not effective either, since these are, in their turn, too wide. These strategies would identify all of the occurrences in examples (4) and (5a)–(5b), but also the following ones and many others alike, which would be false positives:

(6) *They will make progress and will soon come to a conclusion*.
(7) *You should take the money and put it into your account*.

The main assumption behind the work explained here is that MWE-specific morphosyntactic information is helpful for MWE processing in general and MWE identification in particular. As a matter of fact, recent studies [6] have shown that very few word combinations occur in corpora both literally and idiomatically with the very same morphosyntactic features, suggesting that most ambiguities concerning MWEs can be solved by looking at morphology and syntax.

In previous work, in-depth lexical and morphosyntactic information about verb+noun MWEs (VNMWEs) was proven to have a positive impact both in identification [7] and in MT [8]. Detailed data was manually provided in these experiments, and the results obtained using a controlled set of sentences were promising. However, only a few VNMWEs were analysed and, the analysis process being completely manual, the method had a clear scalability problem.

This article describes an improved method where detailed linguistic information about VNMWEs is automatically gathered from corpora, with an aim to reduce manual work and consequently increase the number of analysed VNMWEs. The data acquired by this method was tested on an MWE identification experiment, and both the method and the results are explained here.

The article is organised as follows. After discussing related work, the resources and methodology are described: explanations are given on the two main steps taken, based on a general dictionary. Then, a threefold evaluation if carried out: firstly, the quality of the linguistic information obtained by the method is assessed and commented on; secondly, it is shown how the method was adapted to a different resource; thirdly, it is explained how the gathered data was used for an MWE identification task, and results are given. Finally, some conclusions are drawn and ideas for future work are presented.

## Related work

Two main tasks are usually comprised in MWE processing [5]: discovery and identification. Discovery involves extracting *new* MWEs from corpora in order to create MWE lists (usually with the purpose to create or feed lexicons), while identification aims to find occurrences of previously known MWEs in corpora. Therefore, the input and output of each task is different. For discovery, only a corpus is needed as input, and a list of MWE candidates is obtained as output, automatically extracted from the corpus based on specific models or rules. For identification, on the other hand, an MWE list is also needed as input along with the corpus, and the output is the corpus itself but with MWE annotations. This work focuses on the second of both tasks: identification.

According to Constant *et al*. [5], two NLP applications are relevant to identification: parsing and Machine Translation (MT). Both can benefit from identifying MWEs, since properly identifying MWEs can be helpful to solve ambiguities when parsing, as well as to select the correct translation for a given word combination. For instance, if we consider the words *look* and *up*, distinguishing the occurrences in examples (8) and (9) would help both applications: on the one hand, it would be easier for the parser to tag the words after *look up* as direct object in the first sentence but to treat them as part of a prepositional phrase in the second one; on the other hand, MT systems need to know when to translate the words non-compositionally (example 8) and when compositionally (example 9).

(8) ***Look up*** *the word in the dictionary*. → *buscar* (ES)
(9) *Look up the hill*. → *mirar arriba* (ES)

Conversely, identification can also benefit from parsing, especially when the component words in an MWE are discontiguous (like in examples 4 and 5b).

Several methods have been used to identify MWE occurrences in corpora, including heuristics, disambiguation techniques, machine learning and neural networks. Due to the high level of morphosyntactic flexibility of some MWEs, especially verbal ones [9], efforts have been made to develop sophisticated methods beyond the simplest ones which searched for fixed word sequences only.

The Apertium MT system [10] and the Freeling 3.0 parser [11] are two cases in point. These consider MWE entries as mostly fixed word sequences, but mark part of them as variable and consider all inflected forms of these variable parts. For instance, for the Spanish MWE *tomar en cuenta* ‘take into account’, the verb *tomar* is marked as the variable part, and all possible word forms of this lemma are considered (e.g. *tomarán* ‘they will take’, *tomo* ‘I take’, etc.). This kind of identification system is very precise, although it tends to let many occurrences unidentified, especially when they are discontiguous.

Other authors encode MWE-related constraints in the lexicon and identify only the occurrences that match all constraints [12]. Urizar [13], for instance, comprehensively described 2,207 Basque idioms in the EDBL lexicon [14] by providing information about word order and inflection-related constraints (along with POS and dependency-related information). These data are used within parsing: when the component words of a given MWE are found in text, it is checked whether they match all constraints in the lexicon and, when they do, they are identified as an MWE occurrence.

Nissim and Zaninello [15], on the other hand, take a more generalised approach where variability is described in terms of sequence-based patterns. The Italian expression *degno di nota* ‘worth mentioning’, for instance, would be classified as *flex_fix_fix*, which means that the first lexical component (*degno*) is variable while the rest are not. By using this information for MWE identification, a great increase in precision is obtained, and recall is preserved high for nominal and verbal MWEs, although heavily sacrificed for adjectival and adverbial ones. The method is estimated valuable when a boost in precision is seeked, even if the results obtained are sometimes very similar to the ones obtained by unconstrained searches.

In fact, since variability is considered a key property in the study of MWEs, many authors have looked at specific features related to morphosyntactic variability from a linguistic perspective. In French, Tutin [16] studied 30 frequent VMWEs in terms of five variation types, namely: the plural form, the variation of determiners, the modification of nouns, relative constructions and the passive voice. Like in other related work [9, 17], it was observed that the level of semantic idiomaticity was strongly related to morphosyntactic variability.

The features examined by Tutin are similar to the ones considered in studies about Spanish MWEs. Parra-Escartín *et al*. [18] claimed that most phraseological analyses of Spanish MWEs were not applicable for NLP purposes. Eight tests were proposed in their work, based on previous work by Nunberg *et al*. [19] and by Ramisch [20]. The tests they proposed take into account the possible ellypsis of the verb, the topicalisation of the noun phrase and the pronominalisation of part of the MWE, in addition to the features mentioned by Tutin [16]. Many of these tests are used by Buckingham [21] as well, even if her study focuses on light verb constructions (LVCs) only.

The gradual nature of MWE variability has also been under study, such as in the work by Pasquer *et al*. [22], who presented measures of VMWEs based on variant-to-variant similarity, taking syntactic variability and linear discontiguity into account. The method was evaluated on a French corpus and was observed to have a statistically significant correlation with a linguistic benchmark. Linear similarity was also proved to be useful in VMWE classification and identification.

Another approach to deal with MWE variability is the *MWEtoolkit* [23], which lets users make a number of adaptations on its heuristics: the maximum number of words between MWE components can be specified, the POS of surrounding words can be restricted, etc. Note that this toolkit is language-independent and useful not only for identification but also for discovery.

Other systems are based on disambiguation techniques [24–26] or on machine learning [27, 28]. The first look at the words surrounding a given word combination and mostly rely on distributional semantics, while the second use a number of algorithms to learn from annotated corpora. Most of these only identify contiguous occurrences, although some have developed more sophisticated methods which can deal with discontiguity as well [29].

Research on MWEs have increased considerably in recent years. In view of the importance of MWE processing for NLP tools, the PARSEME COST Action [30] aimed at joining efforts among European researchers interested in computational phraseology. One of the outcomes of this project was the organisation of a shared task on automatic identification of verbal MWEs, which has already held two editions [31, 32]. A multilingual MWE-annotated corpus was released for these, which contains subcorpora in 20 different languages containing annotations of several kinds of verbal MWEs [4].

Seven systems participated in the first edition, most of which were based on machine learning techniques relying on dependency parsing [33]. In the second edition, however, more than half of the seventeen participants used neural networks [34, 35], and there were less which employed traditional machine learning techniques [36]. More details about the shared task will be given later, in the section entitled *Application of the data for MWE identification*.

Note that, for the evaluation of identification systems, the most common measures are precision (P), recall (R) and F-score (F), where:

- P refers to the number of MWE occurrences correctly identified in relation to all occurrences that were identified by the system,
- R refers to the number of MWE occurrences correctly identified by the system in relation to all occurrences that should have been identified,
- and F refers to the harmonic mean of P and R.

## Resources and methodology

The method proposed in this work aims to learn about the morphosyntactic variability of VNMWEs from corpora, in order to use this information to improve MWE identification. The information-gathering method was organised into two main steps.

- A list of Spanish VNMWEs (mostly idioms) was extracted from a general dictionary, and morphosyntactic data about them was gathered from a corpus.
- This information was used to classify VNMWEs into patterns depending on their morphosyntactic variability. Two classification approaches were employed: a heuristic-based one, and another one based on machine learning.

Then, the usefulness of the method was evaluated in three different ways:

- A quality assessment was undertaken, where both the heuristic-based and the machine-learning-based methods were evaluated and compared.
- The method was applied to a list of VNMWEs extracted from a collocations dictionary, to see if the method was useful concerning VNMWEs of a different kind.
- An identification experiment was carried out to see what the real impact of the gathered information is when identifying VNMWEs in text.

The two steps will be described in the following subsections, and details on the evaluation will be given in the next sections.

Note that all data acquired from this process is stored in the *Konbitzul* database [37], which is openly accessible at http://ixa2.si.ehu.eus/konbitzul and can be fully downloaded on CVS from http://doi.org/10.5281/zenodo.3975489.

### Step 1. Extraction of linguistic data from Spanish corpora

In the first step, a list of Spanish VNMWEs was extracted from the *Elhuyar* dictionary (https://hiztegiak.elhuyar.eus), which would be the main input for the whole information-gathering process. This VNMWE list will henceforth be referred to as the *initial list*.

All entries consisting of more than one word in the *Elhuyar* dictionary were POS-tagged by using the Freeling 3.0 parser [11], and the entries tagged as VN combinations were selected to be included in the initial list: a total of 1,205 entries. Note that four kinds of lexical combinations were considered VN combinations: verb+noun, verb+preposition+noun, verb+determiner+noun, verb+preposition+determiner+noun. The *Elhuyar* dictionary being a general dictionary, most of the entries found were idioms, although a number of collocations (especially LVCs) were also included [38].

The aim at this stage was to obtain detailed morphosyntactic information about the occurrences of the VNMWEs in the initial list. Therefore, a monolingual corpus was employed to see how these were used in text. The corpus selected for this purpose was the 15-million-sentence Spanish corpus released for the 2013 Workshop on Machine Translation (available at https://www.statmt.org/wmt13/), which contained texts of various genres, including web-crawled news and documents from the European Parliament and the United Nations. The Freeling 3.0 parser [11] was used to analyse it.

For each of the VNMWEs, the lemmas of the component words were searched for in cases where the noun (and the preposition, when existent) was dependent on the verb. Taking into account the characteristic morphosyntactic aspects of Spanish VNMWEs [18, 21], the following features were looked at:

- Number of the noun phrase (NP): singular (Sing.) or plural (Pl.)
- Presence of determiners in the NP (Det.)
- Definiteness of the NP, in case a determiner was present: definite (Def.) or indefinite (Ind.)
- Presence of modifiers inside the NP (Mod.)
- Alterations in the order of the component words (Ord.)

The information was stored in percentages for all occurrences of each VNMWE candidate. However, many VN combinations can constitute a VNMWE in some sentences but a free expression –or even a different VNMWE– in another one, even when the noun depends on the verb in both cases. This is the case of the verb *dar* ‘give’ and the noun *paso* ‘step’ in Spanish, which can be part of both the VNMWE *dar paso* ‘give way’ (example 10) and the VNMWE *dar pasos* ‘take steps’ (example 11), as well as having coincidental non-idiomatic occurrences such as the one in example (12).

(10) *No es posible* ***dar paso*** *a muchas preguntas hoy*.
no is-it possible give step/way to lots-of questions today
‘It is not possible to give way to many questions today.’
(11) *Vamos a* ***dar*** *un* ***paso*** *transcendental*.
we-will to give one step vital
‘We will take a vital step.’
(12) *Los pasos dieron media vuelta y se marcharon*.
the steps gave half return and refl left
‘The steps turned away and left.’

Based on previous investigations [7], the underlying hypothesis here was that some morphosyntactic features could be especially useful to distinguish between different meanings of the same VN combination. These features were: the syntactic relation (example 13), the possibility to add determiners inside the NP (example 14), and the use of the pronominal form of the verb (example 15).

(13)
  a *Pueden* ***tomar parte*** *en los debates*. → obj.
  they-can take part in the debates
  ‘They can take part in the debates.’
  b *Cada parte tomará las medidas necesarias*. → subj.
  each part will-take the measures necessary
  ‘Each party will take the necessary measures.’
(14)
  a *Esas cuestiones pueden* ***ser de interés*** *para los participantes*. → no det.
  those questions can be of interest for the participants
  ‘Those questions can be interesting for the participants.’
  b *Esto debería* ***ser del interés*** *del cliente*. → det.
  this should be of-the interest of-the client
  ‘This should be of the client’s interest.’
(15)
  a ***Nos damos cuenta*** *de lo ocurrido*. → pronominal
  Pron give account of the happened
  ‘We realise what happened.’
  b *Las autoridades deben* ***dar cuenta*** *de lo ocurrido*. → non-pronominal
  the authorities must give account of the happened
  ‘Authorities must report on what happened.’

We decided that variants differing in these three aspects should be treated separately at Step 1 and merged, when necessary, at Step 2. Information was stored separately for each of these variants, which will henceforth be referred to as *candidates*.

**Fig 1:**
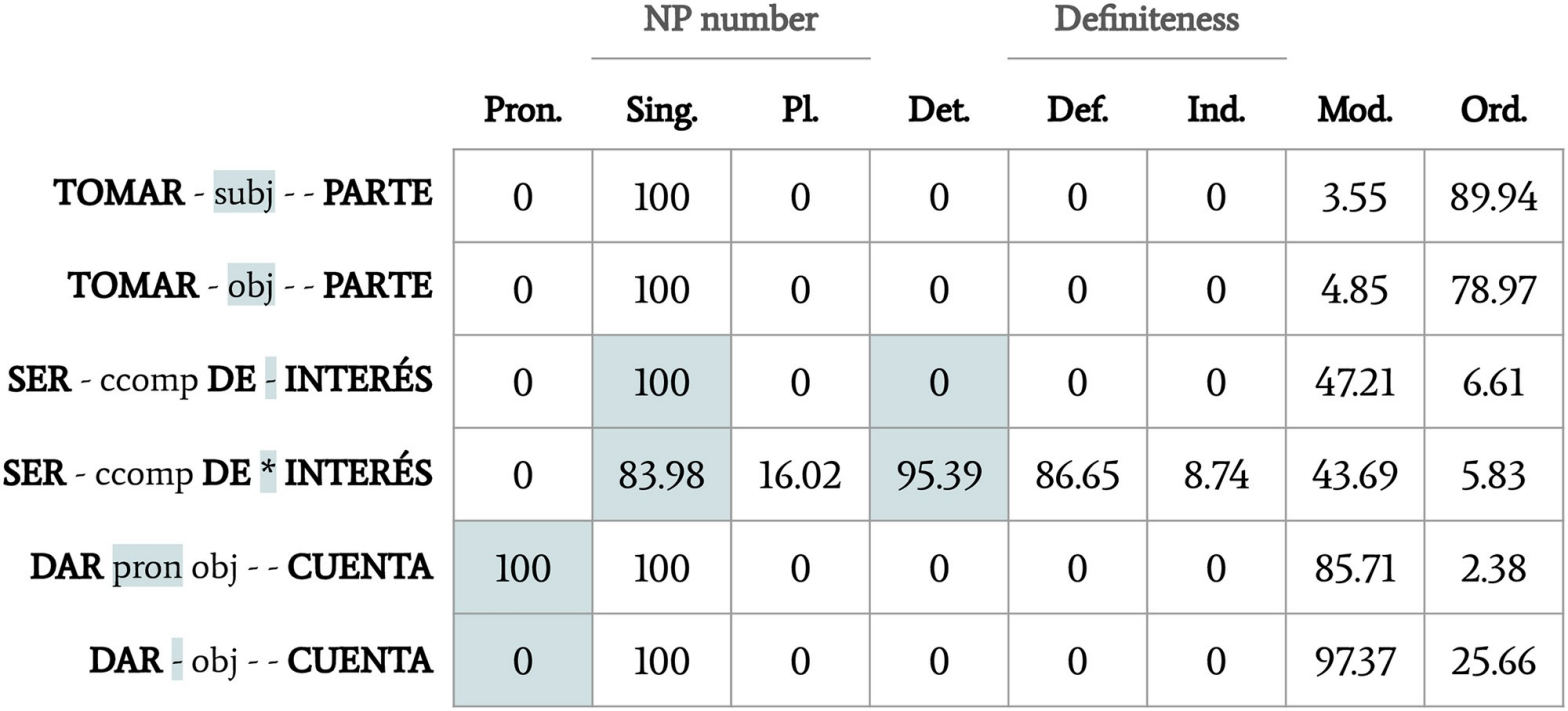
Example of the morphosyntactic table resulting from Step 1. Data in percentages. Candidate keys are organised as follows: VERB, reflexive/pronominal use of the verb (pronominal or non-pronominal: pron|-), syntactic relation (subject, object, circumstancial complement, predicate: subj|obj|ccomp|pred), PREPOSITION, determiners (always present, optional or never present: *|?|-), NOUN.

The features listed above were counted in every occurrence, and a table was created collecting all the data about each candidate (henceforth, the *morphosyntactic table*). Fig 1 shows how information was stored for the combinations in examples (13)–(15).

Many combinations did not occur in the corpus, and others were discarded because their frequency was too low (below 10 occurrences) for the extracted information to be reliable. Finally, 435 candidates were collected along with linguistic data.

### Step 2. Classification of candidates according to morphosyntactic patterns

The second step was to classify the candidates into morphosyntactic *patterns* based on the percentages obtained. The underlying idea here was that, if the very large majority of the occurrences of a given candidate had a given characteristic, e.g. a singular NP with a definite article, the rest of the sentences were unlikely to be relevant to the VNMWE candidate (bearing in mind that divisions were made during Step 1 to help solve ambiguities). Two different approaches were taken for this classification: a heuristic-based one and another one based on machine learning.

#### Heuristic-based approach

In order to indicate from what point on the features should be treated as characteristic for each candidate, two thresholds were manually established and used to turn the percentage values into discrete values:

- If a given candidate *C* had feature *F* in more than 90% of the occurrences, it was assumed that feature *F* was compulsory for candidate *C*.
- If candidate *C* had feature *F* in less than 10% of the occurrences, it was assumed that feature *F* was impossible for candidate *C*.
- If the frequency of feature *F* for candidate *C* was between 10% and 90%, it was assumed that feature *F* was optional for candidate *C*.

For instance, for the candidate *ser de interés* ‘be of interest’ in Fig 1, line 4, the presence of determiner (95.39%) would be considered compulsory, the pronominal use of the verb (0%) would be considered impossible, and the rest of the features would be considered optional.

Except for the syntactic relation, only morphological features were taken into account for the classification into patterns. Information about intra-NP modifiers and alterations in word order was added later, to be used for the identification experiment.

Half of the candidates were used for trials (a total of 218), and the rest (a total of 217) were used to evaluate the quality of the pattern-based classification (see next section). Candidates in the first set were firstly grouped according to their features and, after manually generalising the less productive ones (i.e. the patterns where only one or two candidates fitted), twelve patterns were established. These patterns and their corresponding feature values are specified in Table 1. The values used are *Y* (meaning yes, compulsory), *N* (meaning no, impossible) and *O* (meaning optional).

**Table 1:** Morphosyntactic patterns for the candidates from the Elhuyar dictionary. The first column contains names of patterns.

	Pron.	Sing.	Pl.	Det.	Def.	Ind.
**FREE**	N	O/N	O/N	Y/O/N	Y/O/N	Y/O/N
**PL_NO-DET**	N	N	Y	N	O/N	O/N
**PL_DET_DEF**	N	N	Y	Y	Y	N
**PL**	N	N	Y	Y	Y/O/N	Y/O/N
**SING_NO-DET**	N	Y	N	N	N	N
**SING_DET_DEF**	N	Y	N	Y	Y	N
**SING_DET_IND**	N	Y	N	Y	N	Y
**SING**	N	Y/O	N	Y/O	O/N	O/N
**P_PL**	Y	N	Y	Y/O	Y/O/N	Y/O/N
**P_SING_NO-DET**	Y	Y	N	N	N	N
**P_SING_DET_DEF**	Y	Y	N	Y	Y	N
**P_SING**	Y	Y	N	Y/O	Y/O/N	Y/O/N

Each candidate was classified into its corresponding pattern based on the feature values. Note that, when classifying candidates, the more restrictive patterns were prioritised over the more general ones. This way, if a given candidate fitted in more than one pattern possible, it would finally be classified into the more restrictive one. On the other hand, the candidates where the noun was the subject of a very common verb (*hacer* ‘do/make’, *dar* ‘give’, *tener* ‘have’…) were automatically discarded, since the VNMWEs including these verbs are typically LVCs where the noun is the direct object of the verb [21].

**Fig 2:**
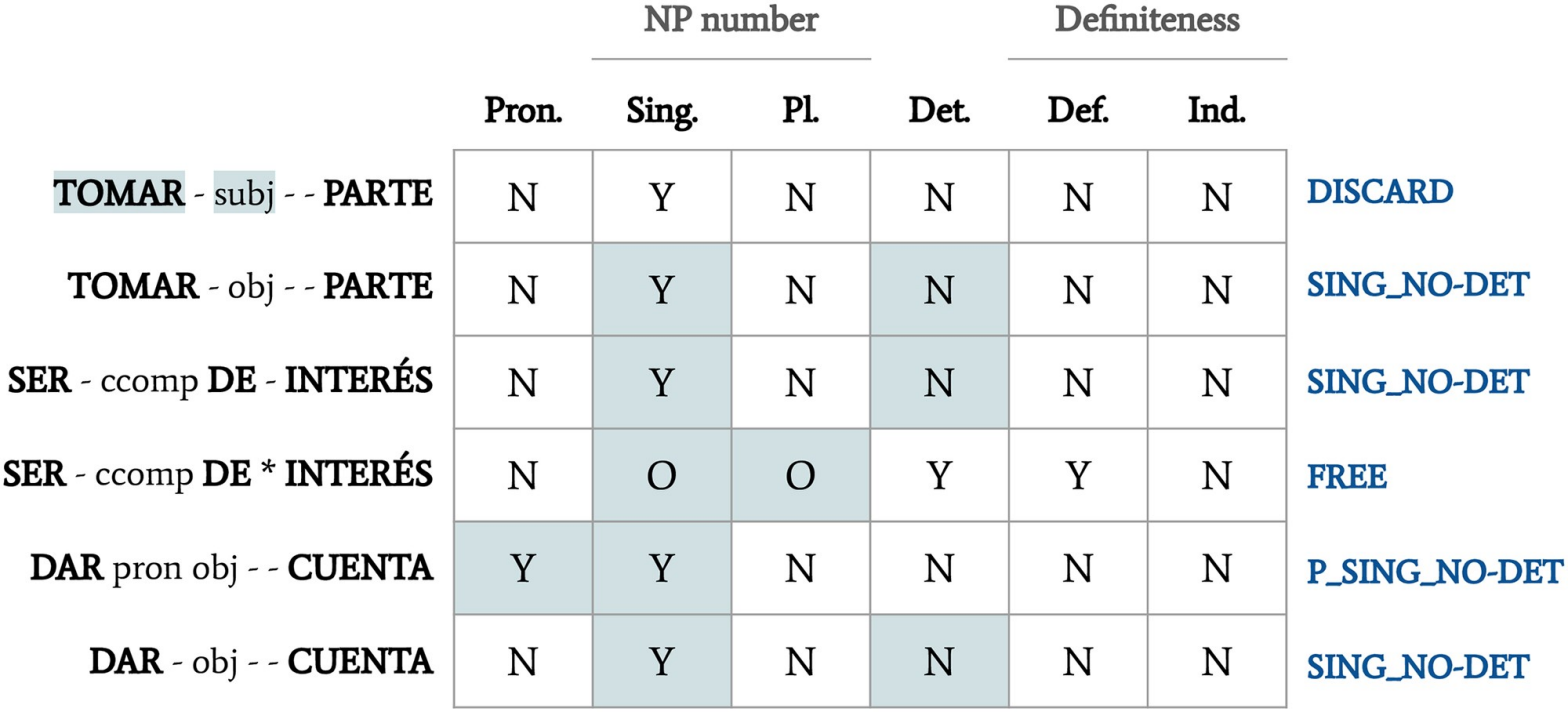
Example of the pattern-based classification using a monolingual corpus. The main features which determine why a given combination is classified in its pattern are marked in blue.

By way of example of the kind of classification done at this stage, Fig 2 shows how the data on Fig 1 evolved, and how the candidates in examples (13)–(15) were classified.

As explained in Step 1, the occurrences of some combinations were divided into more than one candidate, and morphosyntactic information was stored separately for each of them, leading to different pattern-based classifications. In order to verify if the candidates should really be treated as different combinations or if they should be merged into a single one, a parallel corpus was used. The English-Spanish parallel corpus matching the 15-million Spanish corpus used in Step 1 was chosen for this purpose (available at https://www.statmt.org/wmt13/).

The assumption behind this stage was that, if two candidates containing the same word lemmas were usually translated similarly, they were probably morphosyntactic variants of the same combination; however, if they were translated in very different ways, it was likely that they had different meanings, and they should remain separate.

**Fig 3:**
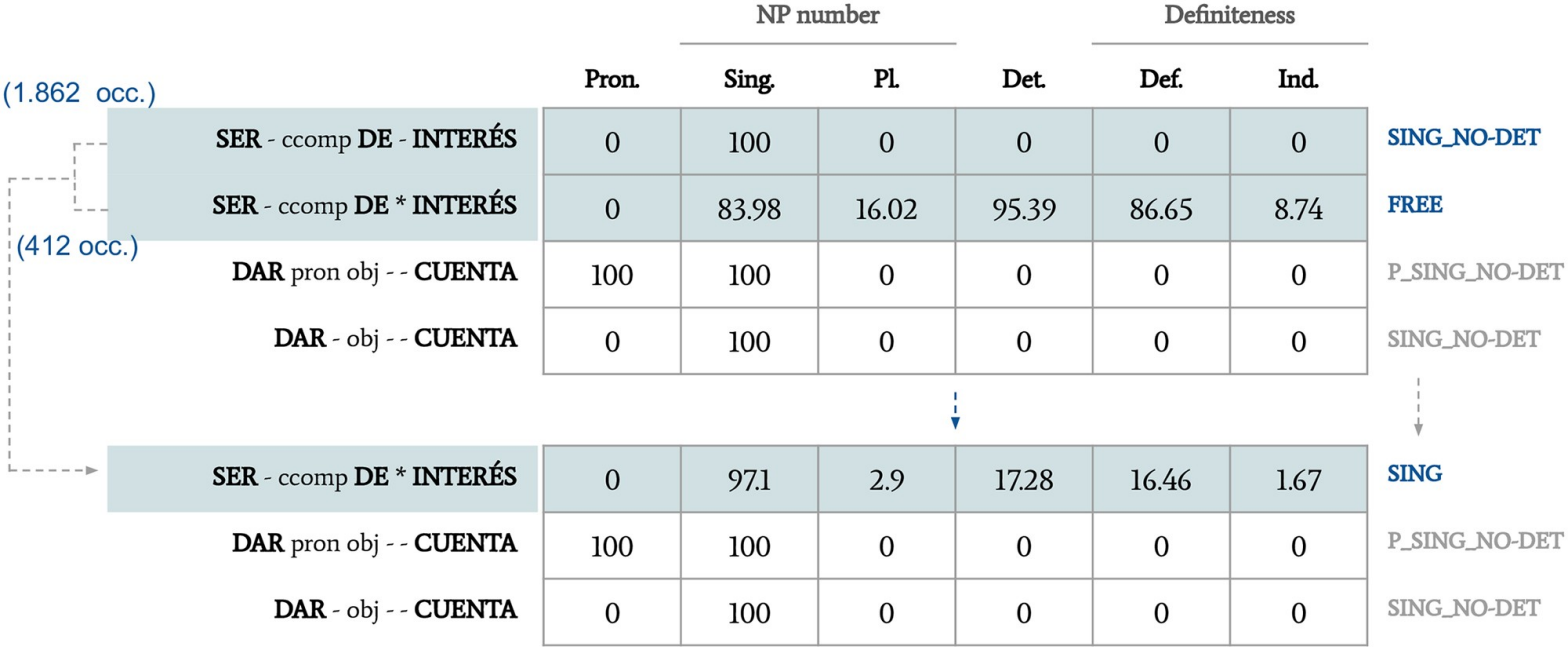
Example of the adjustments on pattern classifications using a parallel corpus.

Word alignments were used to extract possible translations for every candidate, by using the mGIZA tool [39]. Gaps and variations of the word combinations were accepted. Then, the number of shared translations was counted and, if this percentage was higher than a given threshold (35%), which was set manually during trials, the candidates were merged into a single one. Finally, the new merged candidate was re-classified, according to the new combined information (Fig 3).

This is what happened to the combination pairs in examples (14) and (15), previously classified separately (Fig 2). After looking at parallel corpora, it was observed that the amount of translations shared by both candidates in each pair was the following: 15.78% for *dar cuenta* and *darse cuenta* and 64.52% for *ser de interés* and *ser de(l) interés*. Unlike the first pair of candidates, the second pair passed the threshold of shared translations. Thus, the information of this pair of candidates was combined, and the merged candidate was re-classified. Note that, when combining the information, the percentages corresponding to each feature were normalized according to the number of occurrences. Therefore, considering that the number of occurrences of the candidates to be merged in Fig 3 is 1,862+412 = 2,274, the *Sing*. feature is recalculated like this: (1,862*100+412*83.98)/2,274 = 97.1.

#### Machine-learning-based approach

Apart from heuristics, a second approach based on machine learning was also taken to compare results. The whole set of 435 candidates was used, and the features considered were the same ones as in the heuristic-based approach, that is, the ones shown in Table 1.

Since the set of candidates was relatively small, the Leave-One-Out Cross Validation method was selected, which uses one item at a time for testing and the rest for training. The validation process is repeated per each candidate until the whole set is validated. This way, the biggest training set possible is used each time (434 candidates with their corresponding patterns).

Also, due to the small number of candidates available, the algorithms used were the following: Logistic Regression, Random Forest and Naive Bayes (Bernoulli). Results will be given in the next section, along with the ones obtained by the heuristic-based approach.

## Quality assessment of the pattern-based classification

Results of both approaches were calculated to assess the quality of the proposed method, and to see which of the approaches was more appropriate. As pointed out previously, trials for the heuristic-based method were made on half of the candidates (randomly selected), which were manually classified by a single expert annotator into morphosyntactic patterns (see Table 1). Then, the rest of the candidates were also manually assigned morphosyntactic patterns, and this set of 217 candidates was used for testing. Results are shown in Table 2.

**Table 2:** Results of the heuristic-based classification. *Monolingual* and *Parallel* refer to the corpora used. The check mark stands for correct, and the cross for incorrect. The best result is marked in bold.

		Candidates	%
**Monolingual**	✓	118	54.38
	×	99	45.62
**Parallel**	✓	127	**58.53**
	×	90	41.47

As the Table shows, more than half of the candidates were correctly classified on the first round, using a monolingual corpus, and an improvement of around 4 percentage points (around 9 candidates) was obtained on the second round, after using parallel corpora as well. Although the number of candidates used here was quite small, parallel corpora could make a significant difference if a bigger set of candidates is used in the future. Therefore, despite the difference not being very high here, the use of a parallel corpus is advisable.

Concerning the machine-learning-based approach, the whole set was used for validation, as explained in the previous section. Results of the three selected algorithms are shown in Table 3.

**Table 3:** Results of the machine-learning-based classification.

Algorithm	Result
Logistic Regression	**58.18**
Random Forest	48.74
Naive Bayes	37.01

As can be noticed by looking at Tables 2 and 3, the machine-learning-based approach did not outperform the heuristic-based one, which obtained slightly better results overall. However, considering the size of the candidate set, the difference is not statistically significant, so the quality of both approaches can be considered comparable.

It is worth mentioning that a high amount of the erroneously classified candidates were not MWEs but candidates resulting from coincidental occurrences of the lemmas in a given MWE (for a better definition of coincidental occurrences, see [6]). This is the case of example (16b), which is a non-MWE candidate of the VNMWE in (16a), created as a consequence of the divisions made during Step 1.

(16)
  a ***expresar esperanza*** (lit. express hope) → obj.
  b *expresar esperanza* (lit. express hope) → subj.

The division of candidates was still positively valued as a disambiguation strategy. Nevertheless, for future work, it would be convenient to develop a refined division procedure, perhaps including a more sophisticated way of discarding redundant candidates, since this overgeneration can affect the quality of the whole method negatively. A possible way to do this is to automatically discard all candidates where the noun is the subject of the verb, since VNMWEs of this kind are very scarce. On the other hand, it would be interesting to try the same method with a different parser, in order to see what the impact of the quality of the parser is on the whole process.

### Adapting the method to another resource: From a general to a collocations dictionary

Once the quality of the information-gathering method was assessed based on the Elhuyar dictionary, a second source was used to see if the method could be appropriate for other kinds of VNMWEs. The DiCE dictionary of Spanish collocations [40] was selected for this purpose. This dictionary differs from the Elhuyar one in two main aspects:

- As the name of the dictionary indicates, the combinations considered in DiCE are all collocations, many of them being of the LVC subtype. In Elhuyar, mostly idioms and LVCs were included, but not any other kind of collocation.
- Entries in DiCE contain nouns only, and collocates are listed as subentries organised by their grammatical category. Therefore, as opposed to the VN combinations gathered from Elhuyar, the VN combinations extracted from DiCE are all combinations consisting of only a verb and a noun, since no prepositions and determiners are specified. Note, however, that this does not mean that the VN collocations are never used with a preposition or a determiner in-between. For instance, concerning the collocation *ser del agrado (de alguien)* ‘be of somebody’s liking’, the entry in DiCE is the noun *agrado* ‘liking’, and the verb *ser* ‘be’ is listed as a subentry; the preposition *de* ‘of’ is not specified.

After having extracted all VN entries from DiCE, the 500 most frequent ones [41] were selected to be used as an input for the information-gathering process. The entries already existing in Elhuyar were discarded, and an initial list of 437 VN collocations was created.

Due to the different nature of the VN combinations in the Elhuyar-based and DiCE-based initial lists, some adjustments had to be made on the information-gathering process. In Step 1, apart from the divisions illustrated by examples (13)–(15), one more division was done so as to get information about prepositions from the corpus.

(17)
  a *No lo* ***dejaremos a*** *un* ***lado***.
  no him we-will-leave to one side
  ‘We will not let him aside.’
  b *No lo* ***dejaremos de lado***.
  no him leave of side
  ‘We will not let him aside.’

Due to these duplications, 544 candidates resulted from the 437 collocations in the initial list. A Morphosyntactic Table like the one shown in Fig 1 was created as a result of Step 1, which was then used to classify the candidates into patterns in Step 2. Since the heuristic-based approach was followed, half of the candidates were used for trials, and the rest for testing.

The same patterns created for the candidates in Elhuyar were tried on the training set at first, but it was soon observed that many of them had too many restrictions to be applied to collocations, since these are typically more flexible morphosyntactically [17]. Therefore, we decided to reduce the patterns to five, as shown in Table 4.

**Table 4:** Morphosyntactic patterns for the candidates from the DiCE dictionary. The first column contains names of patterns.

	Pron.	Sing.	Pl.	Det.	Def.	Ind.
**FREE**	N	O/N	Y/O/N	Y/O/N	Y/O/N	Y/O/N
**SING_NO-DET**	N	Y	N	N	N	N
**SING_DET_DEF**	N	Y	N	Y	Y	N
**SING_DET_IND**	N	Y	N	Y	N	Y
**SING**	N	Y/O	N	Y/O	O/N	O/N

As can be noticed, seven patterns were discarded in all, due to their lack of relevance to the candidates from DiCE. This was done manually after classifying all candidates in the training set, since no (or very few) candidates were classified in them. The discarded patterns were: (a) those collecting the combinations used in the pronominal form only and (b) those collecting the combinations used in the plural form only.

After applying the whole methodology, the results collected in Table 5 were obtained. As can be noticed, results are very similar to the ones obtained by using the Elhuyar dictionary, which confirms that the method is easily adaptable to resources of different kinds.

**Table 5:** Results of the method based on the DiCE dictionary. *Monolingual* and *Parallel* refer to the corpora used. The best result is marked in bold.

		Candidates	%
**Monolingual**	✓	148	54.42
	×	124	45.59
**Parallel**	✓	161	**59.19**
	×	111	40.81

## Application of the data for MWE identification

In order to test whether the information gathered by the method was useful to improve MWE identification, an experiment was undertaken. The Spanish part of the PARSEME multilingual corpus [4] was used, so that our results could be compared to the results from the PARSEME shared task on automatic identification of verbal MWEs, edition 1.1 [31].

However, it must be taken into account that the criteria followed to annotate this corpus were not completely compatible with ours. More precisely, the MWEs annotated in the PARSEME corpus differ from the MWEs considered in our information-gathering process in two main aspects:

- MWEs composed by all kinds of grammatical categories are annotated, such as verb+adjective and verb+preposition, not only VNMWEs.
- Collocations which are not LVCs are excluded.

With a view to carrying out an experiment as comparable as possible to the PARSEME shared task, a few adjustments had to be made both on the VNMWE dataset and on the corpus. Since the PARSEME multilingual corpus was released with not only MWE tags but also morphosyntactic information (based on Universal Dependencies), the preparation of the corpus was quite simple. Only the MWE tags in which the component words were a verb and a noun (sometimes also with a preposition or determiner) were considered, omitting the rest and creating an adapted corpus of 5,515 sentences and 662 MWE tags, distributed as follows: 355 in the Train corpus, 136 in the Development corpus, and 171 in the Test corpus.

On the other hand, so as to avoid problems coming from disparities in the conception of MWEs according to PARSEME and the source dictionaries we used, only the word combinations annotated in the corpus were looked for. The information-gathering method was applied to all 491 VNMWE tags in the Train and Development corpora, and a new dataset was built. Some of the tags (239 in all) could not be classified by the method because of their low frequency; the remaining 252 were found to be occurrences of 95 VNMWEs, and were automatically classified according to patterns, following the steps described in the previous section.

Then, the overall identification strategy was the following:

1. The component lemmas of the automatically classified VNMWEs were searched for, and the restrictions corresponding to their morphosyntactic patterns were applied in order to discard non-MWE occurrences. Morphosyntactic information provided by a parser was used to apply restrictions, like in [7].
2. The VNMWEs which could not be automatically classified were treated in two different ways:
  - As fixed word sequences where the only variation possible was verb inflection. The verb lemma was looked for, but the rest of the components needed to be in the same word form as in the corpus tag. All component words needed to be contiguous and respect the order in the corpus tag.
  - As completely flexible combinations. The lemmas of the component words were looked for, in any order and word form.

Two baselines were also created to be used for comparison, where only the A and B methods were applied, without any pattern-based information. Henceforth, we will refer to the methods proposed above as *Pattern-based-A* and *Pattern-based-B*, and the two baselines as *Baseline-A* and *Baseline-B*.

When creating the initial list of VNMWEs to be used as input for the whole information-gathering process and for the identification experiment, the list of VNMWEs was based on the tags in the Train and Development parts of the PARSEME corpus. As for testing, the Test part of the corpus was used.

Results are shown in Table 6. As it was noted above, the pattern-based identification methods proposed in this paper were compared to two baselines which use no VNMWE-specific information, in order to better measure the impact of using morphosyntactic information related to VNMWE variability.

**Table 6:** Results of the identification experiment. The best scores in P, R and F are marked in bold.

	P	R	F
**Pattern-based-A**	**0.74**	0.31	**0.52**
**Pattern-based-B**	0.61	0.33	0.47
**Baseline-A**	0.73	0.20	0.47
**Baseline-B**	0.61	**0.37**	0.49

As can be noticed, the best score was obtained by method *Pattern-based-A*, with an F score of 0.52; precision was higher than in the rest of the methods (0.74), and recall was acceptable as well (0.31), although not as high as the one obtained by *Baseline-B*. However, it must be noted that all methods had quite a low recall, which was quite general among the 17 systems which participated in the PARSEME shared task [31] (Table 7), mostly because a lot of VNMWEs in the Test corpus did not occur in the Train and Development parts.

**Table 7:** Results of the PARSEME shared task edition 1.1, in Spanish and in all 20 languages. Apart from the average score obtained, the lowest and highest scores are also shown between brackets.

	P	R	F
**ES**	0.19 (0.00-0.32)	0.33 (0.00-0.49)	0.23 (0.00-0.38)
**All languages**	0.36 (0.00-0.68)	0.29 (0.01-0.53)	0.31 (0.00-0.54)

Although the recall of *Pattern-based-A* is a bit under the average recall in Spanish, its precision is much higher, which led it to achieve an F score of 14 points higher than the best-performing system in the Spanish part of the shared task [34]. The best recall was obtained by *Baseline-B*, but its precision being much lower, F-score was not as high.

While it is true that these results are not completely comparable to the ones in the PARSEME shared task, since verbal MWEs other than VNMWEs were also considered there, category-based results suggest that VNMWEs are exactly one of the most difficult kind of MWE to identify. As a matter of fact, MWEs composed of a verb and a noun fall into three of the verbal MWE categories considered in the PARSEME corpus: LVC.full, light verb constructions where the verb is fully light; LVC.cause, light verb constructions where the verb adds a causative meaning; and VID, verbal idioms. These are indeed the categories where the lowest F scores were obtained (Table 8).

**Table 8:** Spanish results of the PARSEME shared task edition 1.1 by MWE category. Only the three categories including VNMWEs are shown in the Table. Apart from the average score obtained in each category, the lowest and highest scores are also shown between brackets.

	P	R	F
**LVC.cause**	0.13 (0-100)	0.02 (0-0.21)	0.03 (0-0.30)
**LVC.full**	0.17 (0-0.48)	0.14 (0-0.41)	0.13 (0-0.33)
**VID**	0.14 (0-0.46)	0.08 (0-0.23)	0.10 (0-0.31)

Therefore, the main conclusion is that the proposed method outperforms all systems which participated in the Spanish part of the PARSEME shared task edition 1.1, which confirms the usefulness of the information-gathering method for the identification of MWEs. Additionally, it must be taken into account that the dataset used here was the output of a completely automatic analysis process, with no manual adjustment on the morphosyntactic patterns obtained. If manual checks were performed, results would probably be even better.

## Conclusion

A method to automatically gather VNMWE-specific linguistic information from corpora was described in this paper. Morphosyntactic features were especially looked at, and these were then evaluated in terms of an MWE identification experiment. The linguistic information resulting from the whole process is now stored in a publicly available database, *Konbitzul* (http://ixa2.si.ehu.eus/konbitzul/?lang=en), which was specifically created for this purpose.

In MWE identification, an F score of 0.52 was obtained using the Spanish part of the PARSEME corpus, released for the PARSEME shared task on automatic identification of verbal MWEs. This score is 14 points higher than the best-performing system (among 17) in edition 1.1 of the shared task.

For future work, the method would benefit from some improvements, especially in order to automatically discard more non-MWEs from the datasets. The use of different corpora (i.e. of specialised text) would also be helpful to see how the use of MWEs changes between domains. Finally, it would be interesting to keep developing the method by including MWEs other than VN combinations, as well as by testing it in languages other than Spanish.
